# Editorial: Vascular Health: The Endothelial Perspective in Regulation of Inflammation and Injury

**DOI:** 10.3389/fphys.2021.732234

**Published:** 2021-08-06

**Authors:** Shampa Chatterjee, Silvia Lacchini, Wolfgang Jungraithmayr, Felix W. Wehrli

**Affiliations:** ^1^Department of Physiology, Institute for Environmental Medicine, University of Pennsylvania Perelman School of Medicine, Philadelphia, PA, United States; ^2^Department of Anatomy, Institute of Biomedical Sciences, University of São Paulo, São Paulo, Brazil; ^3^Department of Thoracic Surgery, Faculty of Medicine, Medical Center - University of Freiburg, Freiburg, Germany; ^4^Radiologic Science, Biochemistry and Biophysics, University of Pennsylvania Perelman School of Medicine, Philadelphia, PA, United States

**Keywords:** endothelium, oxidative stress, inflammation, vascular dysfunction, hyperproliferation, autophagy, transplant signaling, imaging–radiology

This special Research Topic of Frontiers in Physiology collates 16 (review and original research) articles on the endothelium (the cell layer that lines blood vessels) and other components of the vascular system. Over the past decade, it has become increasingly clear that the vascular network is not a mere conduit for blood and the endothelium is not a “wallpaper” of the vascular network; rather both are dynamic entities that sense and respond to mechanical and chemical stimulation from the microenvironment. Both endothelial cells and vascular tissue (comprising of endothelial and smooth muscle layer and other cells such as inflammatory/immune cells) are equipped with complex molecular machinery that initiate and amplify signaling processes leading to physiological and pathophysiological changes. These changes range from vasodilation, vasoconstriction and angiogenesis to inflammation, leakage, injury, and vascular remodeling and form the basis of vascular health, disease and other etiologies.

Endothelial signaling initiated by various stimuli involve reactive oxygen species (ROS) induced signaling events, calcium influx (via various Ca^2+^ channels), activation of mitogen-activated protein (MAP) kinases, induction of cellular adhesion molecules (CAMs) which lead either to activation of downstream inflammation moieties (such as the NLRP3 inflammasome) or to modification of proteins that facilitate endothelial cell function. Collectively, these changes can alter vascular cell-cell junctions, vessel structure, and function. For instance, endothelial cells of a donor organ as the interface between the transplanted organ (graft) and the immune cells of the host, serve as the first target for host (recipient) immune cells post-transplant. Kummer et al. review how the sensing of a “foreign” entity by the human leukocyte antigen (HLA) I and II-recognizing natural killer (NK) cells, macrophages, and T-cells lead these cells to attack the endothelial layer. The damaged endothelial cells produce chemokines, express CAMs and drive a pro-inflammatory microenvironment. Inflammatory cells (macrophages, leukocytes, T-cells etc.) are recruited into this environment and transmigrate from the bloodstream across the endothelial monolayer into the vessel wall, causing vascular damage. Vascular damage initiates repair mechanisms whereby migration and continued proliferation of vascular smooth muscle cells occur in the vascular structures causing fibrous intimal hyperplasia, which ultimately leads to transplant failure. Thus, endothelial preservation, as proposed by Jungraithmayr can be a promising therapeutic strategy for the protection of the endothelium against ischemia and reperfusion (i.e. maneuvers associated with transplant). Therefore, various receptors and signaling pathways that are well-recognized to be activated upon transplantation (ROS, MAP kinases, CAMs, angiotensin-converting enzyme) can be blocked in order to facilitate endothelial preservation against injury.

ROS production and calcium influx are pivotal cellular events that drive inflammation signaling. ROS production in endothelial cells is tightly regulated by certain receptors including the Cystic Fibrosis Transmembrane Conductance Regulator (CFTR). As reported by Khalaf et al., CFTR plays a role in endothelial oxidative damage by controlling ROS production. In naïve endothelial cells, ROS production is low but increases following inhibition of CFTR activity. ROS has been proposed as an upstream inducer of the NLRP3 inflammasome, a well-characterized platform of multiple subunits (NLRP3, ASC, pro-caspase) that cause caspase and interleukin (IL)-1β, IL-18 release, leading to inflammation induced cell death (pyroptosis). Thus, various pathologies or agents that drive NLRP3 activation can also lead to endothelial inflammation and injury. As reported by Pereira et al. in a murine model of diabetes, wild-type mice showed reduction in endothelium-dependent vasodilation, increased vascular ROS generation, caspase-1, and IL-1β activation. These changes were not observed in Nlrp3^−/−^ mice, which appear to be protected. Paul et al. review the mechanisms underpinning the links between periodontal disease (PD) and diabetes and cardiovascular disease. ROS and oxidative stress may also play a role in amplification of bacterial driven inflammation by triggering pathological processes in distant organs. A two-way association between PD and other health conditions presumably arises via ROS and intermediate inflammation signaling. Thus, blocking these intermediate inflammation-signaling events (such as activation of the NLRP3 inflammasome, cytokines, chemokines) can facilitate endothelial preservation. Modification of proteins also serve as intermediate signals that can alter inflammatory mediators/response. Silva et al. show that in a murine model of sepsis (which results in a systemic inflammatory state that accompanies endothelial dysfunction), inflammation is reduced by acylation of proteins by O-linked N-acetylglucosamine (O-GlcNAcylation); indeed, increase in O-GlcNAc reduces systemic inflammation and cardiovascular dysfunction, indicating the therapeutic potential of this pathway.

Like ROS, calcium ions (Ca^2+^) are prominent signaling molecules that participate in various physiological functions, such as vasodilation, cell migration, growth and apoptosis. In naïve cells, mitochondria sequester and release Ca^2+^ via mitochondrial calcium uniporter complexes. While oxidative stress or other deterrent stimuli, increase mitochondrial calcium overload leading to apoptotic cell death, the calcium uniporter complexes regulate prosurvival mechanisms such as autophagy for endothelial protection. Natarajan et al. report that with *in vitro* ischemia (achieved by oxygen-glucose deprivation) endothelial cells downregulate the mitochondrial calcium uniporter complex 1 (MCUR1), but that the resultant autophagic flux is insufficient to protect ischemic endothelial cells and an additional autophagic flux (by serum starvation of endothelial cells) is required for cell protection indicating that therapeutic strategies can involve targeting multiple mechanisms of endothelial protection.

How does the vasculature respond to inflammation, oxidative damage and injury? A major response to vascular injury is a hyperproliferative response of vascular smooth muscle cells (VSMC). This gives rise of hyperplasia which leads to vessel remodeling. This is detrimental specifically in the context of maneuvers such as percutaneous coronary intervention where balloon angioplasty to remove arterial blockage is followed by hyperplasia (VSMC proliferation) that reinstates the blockage (restenosis). Restenosis can potentially be alleviated by blockade of vascular signaling that drives hyperproliferation. Toward that goal, Li et al. use osthole (7-methoxy-8-isopentenoxy-coumarin) a pharmacologically active component of *Cnidium monnieri* (L.) Cusson and report that it alleviates hyperplasia in a rat model of balloon angioplasty by targeting the NF-κB and TGF-β1/Smad2 signaling pathways that play a role in facilitating VSMC proliferation. Vascularization also has implications in preeclampsia, as reviewed by Raguema et al. Abnormal placental vascularization presumably arises from chronic hypoxia due to reduced placental blood flow leading to fetal growth retardation and later to pre-eclampsia.

Another response is the disruption of the endothelial cell barrier. Indeed, as Baruah et al. review intrinsic defects within endothelial cells from the earliest developmental time points, the disruption of the blood-brain barrier (BBB) can be the cause for posttraumatic epilepsy and other similar etiologies. Under control conditions, the BBB is maintained via tight endothelial cell-cell junctions, but these can be loosened by inflammation and oxidative damage. These changes lead to vascular abnormalities and autonomously support the development of hyperexcitability and epileptiform activity. Thus, BBB protection by alleviation in inflammation or by repair of junctional proteins can be a potential therapeutic strategy in epilepsy control.

Vascular inflammation also affects cellular signaling of bone. Epsley et al. review the effect of inflammation on bone modeling, which is a continual process to renew the adult skeleton through the sequential growth and resorption processes of bone cells (osteoblasts and osteoclasts). Nuclear factor RANK, an osteoclast receptor, and its ligand RANKL, expressed on the surface of osteoblasts, result in coordinated control of bone remodeling. Inflammation skews this process toward resorption via the interaction of inflammatory mediators and their related peptides with osteoblasts and osteoclasts, as well as other immune cells, to alter the expression of RANK and RANKL.

Changes in endothelial cell signaling that transiently or chronically impair endothelial function, vascular reactivity, and oxygen metabolism can be evaluated non-invasively by quantitative magnetic resonance imaging (MRI). Englund and Langham review the use of MRI for vascular parameters via dynamic quantification of blood flow and oxygenation under conditions of ischemia or exercise and showcase this application for detection of endothelial dysfunction in clinical (cardiovascular disease) and sub-clinical settings (chronic smokers, non-smokers exposed to e-cigarette aerosol etc.). Wehrli et al. review the ability of MRI to detect vascular function. When paired with endothelial cell signaling assays, these “MRI-biochemical pairing” methods highlight the fact that changes at the cellular level (i.e. microvascular endothelial signals) translate into altered vascular function at the macro level. Thus, MRI measures of vascular function may ultimately be used to complement the standard clinical metrics of vascular function and provide additional insight into efficacy of drugs for improvement of vascular function. Similarly, as reviewed by Mayer et al. endothelial signaling at cellular and molecular level, that contribute to atherosclerosis can be detected by non-invasive monitoring of calcification, a complex process facilitated by endothelial signaling that leads to transition of endothelial cells to osteoblast-like cells. Mayer et al. review existing literature on 18F-fluorodeoxyglucose (FDG) and 18F-sodium fluoride (NaF) positron emission tomography (PET) in assessing atherosclerosis via microcalcification and characteristic plaque buildup. Since the initiating event of atherogenesis is inflammation, the pathogenesis manifests in increased metabolic activity, detectable by FDG. At a later stage, atherosclerotic plaques become calcified. Calcification entails incorporation of calcium hydroxy apatite into the vascular walls. Injection of NaF as a tracer leads to partial exchange of hydroxyl ions by fluoride, therefore allowing direct detection of plaque constituents. Pourfathi et al. review the role of PET and hyperpolarized ^13^C MRI imaging to detect the various stages of pulmonary inflammation. The most common PET tracer for metabolic imaging is FDG. A hallmark of inflammatory lung disease is increased glycolysis manifesting in enhanced FDG PET activity. An alternative strategy, which is at an early stage of development, is to quantify the formation of lactic acid by means of hyperpolarized carbon-13 spectroscopic imaging. The carbonyl carbons of lactic acid and its exogenously administered metabolic precursor (pyruvate) have characteristic chemical shifts allowing their distinction.

In conclusion, endothelial signaling has an impact on overall health. Under conditions where these signals lead to remodeling that affects homeostasis, interventions (such as employing respiratory techniques as reported by Fetter et al. can alter blood flow and improve vascular function) or inhibition/blockade of ROS or NLRP3 or inflammatory and metabolic pathways may be beneficial to restore vascular function.

## Author Contributions

SC drafted the editorial. All authors edited the article and approved the submitted version.

## Conflict of Interest

The authors declare that the research was conducted in the absence of any commercial or financial relationships that could be construed as a potential conflict of interest.

## Publisher's Note

All claims expressed in this article are solely those of the authors and do not necessarily represent those of their affiliated organizations, or those of the publisher, the editors and the reviewers. Any product that may be evaluated in this article, or claim that may be made by its manufacturer, is not guaranteed or endorsed by the publisher.

